# Cancer fear and fatalism among ethnic minority women in the United Kingdom

**DOI:** 10.1038/bjc.2016.15

**Published:** 2016-02-11

**Authors:** Charlotte Vrinten, Jane Wardle, Laura AV Marlow

**Affiliations:** 1 Cancer Research UK Health Behaviour Research Centre, Department of Epidemiology and Public Health, UCL, Gower Street, London WC1E 6BT, UK

**Keywords:** cancer, fear, worry, fatalism, ethnicity, ethnic group, screening, early detection

## Abstract

**Background:**

Cancer fear and fatalism are believed to be higher in ethnic minorities and may contribute to lower engagement with cancer prevention and early detection. We explored the levels of cancer fear and fatalism in six ethnic groups in the United Kingdom and examined the contribution of acculturation and general fatalism.

**Methods:**

A cross-sectional survey of 720 White British, Caribbean, African, Indian, Pakistani, and Bangladeshi women (120 of each) was conducted. Three items assessed cancer fear and two cancer fatalism. Acculturation was assessed using (self-reported) migration status, ability to speak English, and understanding of health leaflets; general fatalism with a standard measure.

**Results:**

Relative to White British women, African and Indian women were more fearful of cancer, Bangladeshi women less fearful, and Pakistani and Caribbean women were similar to White British women. Cancer fatalism was higher in all the ethnic minority groups compared with White British women. Less acculturated women were less likely to worry (ORs 0.21–0.45, all *P*<0.05) or feel particularly afraid (ORs 0.11–0.31, all *P*<0.05) but more likely to feel uncomfortable about cancer (ORs 1.97–3.03, all *P*<0.05). Lower acculturation (ORs 4.30–17.27, *P*<0.05) and general fatalism (OR 2.29, *P*<0.05) were associated with the belief that cancer is predetermined.

**Conclusions:**

In general, cancer fear and fatalism are more prevalent among ethnic minority than White British women and even more so in less acculturated ethnic minorities. This may affect their participation in cancer prevention and early detection.

Cancer fear and fatalism are associated with lower uptake of cancer screening (Straughan and Seow, 1998; Austin *et al*, 2002; Robb *et al*, 2008; Vrinten *et al*, 2015) and may contribute to delayed presentation of cancer symptoms (Robb *et al*, 2009; Beeken *et al*, 2011; Bergamo *et al*, 2013; Jones *et al*, 2014; Balasooriya-Smeekens *et al*, 2015; Lyratzopoulos *et al*, 2015). It is therefore increasingly recognised that fatalistic beliefs and emotional factors such as cancer fear need to be addressed in public health campaigns to successfully change behaviour (Niederdeppe and Levy, 2007; Robb *et al*, 2009). Messages such as ‘cancer screening saves lives', ‘finding cancer early makes it more treatable', and ‘your mind will be put at rest (if you see your doctor about a symptom)' are becoming core components of cancer awareness campaigns, such as the ‘Be Clear on Cancer' campaigns in the United Kingdom (Centers for Disease Control and Prevention, 2009; Public Health England, n.d.).

Cancer fear is defined as a negative emotional reaction to the threat of cancer (Hay *et al*, 2005). Cancer fatalism is the belief that a cancer diagnosis is a matter of fate and therefore beyond the individual's control (Straughan and Seow, 1998) or the belief that death is inevitable when cancer is present (Powe and Finnie, 2003), although some measures include both (e.g., the Powe Fatalism Index; Powe, 1995). Cancer fear and fatalism are distinct, but associated, constructs: cancer fear refers to the affective response to the threat of cancer, while cancer fatalism refers to cognitions about cancer. The two are moderately correlated; more fatalistic attitudes towards cancer tend to be associated with being more fearful of cancer (Miles *et al*, 2008; Beeken *et al*, 2011).

Women and those with lower levels of education tend to have higher levels of cancer fear and fatalism (Powe and Finnie, 2003; Consedine *et al*, 2004b; Kudadjie-Gyamfi *et al*, 2005; Vrinten *et al*, 2014). Associations with age are less consistent, with higher levels of fear in those who are younger (Consedine *et al*, 2004b; Kudadjie-Gyamfi *et al*, 2005; Vrinten *et al*, 2014) and higher fatalism in those who are older (Powe and Finnie, 2003). Cancer fear and fatalism also tend to be higher in ethnic minority groups. For example, data from a large population-based survey in the United States showed that Latino, Black, and Asian minority groups were more fatalistic about the preventability of cancer than those from White non-Latino backgrounds (Ramírez *et al*, 2013). These differences remained significant for the Latino and Asian groups after controlling for age, gender, and level of education. A number of qualitative studies have suggested that higher levels of cancer fear and fatalism may be one explanation for ethnic inequalities in cancer screening uptake and delayed symptom presentation (Randhawa and Owens, 2004; Thomas *et al*, 2005). This suggestion also gained support from several quantitative studies (Austin *et al*, 2002; Johnson *et al*, 2008; Bergamo *et al*, 2013). For example, a large population-based study in the United Kingdom showed that those from Asian and Black backgrounds were more fearful and fatalistic about bowel cancer than those from White ethnic backgrounds, and this was associated with lower uptake of colorectal cancer screening (Robb *et al*, 2008).

However, levels of cancer fear and fatalism are not the same across all ethnic minority groups (Jacobson, 1999). In a large study conducted in the United States, up to 62% of Latinos believed that cancer was not preventable as against 33% of Asians, 29% of Blacks, and 22% of Whites (Ramírez *et al*, 2013). Another large US-based study found that breast cancer worry was highest in Haitian and Dominican women, followed by Eastern European, English Caribbean, and African American women, whereas European American women had the lowest levels of worry (Consedine, 2012). A similar pattern was observed for breast cancer fatalism in this study.

The differences in levels of cancer fear and fatalism across ethnic groups suggest that other cultural factors, such as language spoken and migration status, may have a role in explaining variations in these variables. For some ethnic groups, language barriers may impede understanding of the preventive purpose of cancer screening tests, which may increase levels of cancer fear when offered one (Meana *et al*, 2001; Austin, 2009). Language barriers may also have a role in perpetuating fatalistic beliefs: 62% of Spanish-speaking Latinos believed that cancer was not preventable, *vs* 40% of English-speaking Latinos (Ramírez *et al*, 2013). In addition, in some cultures, the word ‘cancer' itself is an object of fear, and it is instead referred to as ‘the big C' or ‘that disease' (Taha, 2012; Jones *et al*, 2014). This may lead to reluctance to read public health messages about cancer and may also perpetuate cancer fear and fatalistic beliefs.

A fatalistic outlook on life in general may also shape fatalistic beliefs about cancer (Straughan and Seow, 1998; Powe and Finnie, 2003). A general sense of fatalism may be more prevalent among certain ethnic groups, for whom cycles of poverty, unemployment, racism, and discrimination could have fostered the belief that events in life are beyond the individual's control (Powe and Finnie, 2003). This may lead to cancer fatalism when a lack of health-care access subsequently leads to poor health outcomes for cancer (Powe and Finnie, 2003). In addition, some ethnic groups are more likely to believe that God is in control over what happens in life, and this belief may also extend to whether or not someone will get cancer (Koffman *et al*, 2008).

In the present study, we explore cancer fear and fatalism among women from six different ethnic backgrounds living in the United Kingdom. We also explore the role of language spoken, migration status, and general fatalistic beliefs in explaining cancer fear and cancer fatalism among ethnic minority women.

## Materials and methods

### Participants

Participants were recruited via the commercial sampling service Ethnic Focus, which uses quota sampling to recruit participants from ethnic minority backgrounds across England. We commissioned them to recruit 120 women aged 30–60 years from each of the following ethnic backgrounds: Indian, Pakistani, Bangladeshi, African, Caribbean, and White British, for a total sample size of 720 women. Data for the present study come from a wider study on attitudes to cancer and cervical cancer screening (Marlow *et al*, 2015), a cancer screening programme which is implemented in the United Kingdom via GP practices. However, Chaturvedi and McKeigue (1994) argue that recruitment of ethnic minority samples into epidemiological studies via GP practices may be problematic because of higher proportions of people not registered with GP practices or contact details registered with GPs not being up-to-date, especially in inner city areas, where large proportions of those from ethnic minority backgrounds tend to live. We therefore opted for a commercial sampling service with extensive experience in recruiting respondents from ethnic minority backgrounds to make sure that recruitment into the study was not dependent on being registered with a GP and the GP contact details of potential participants being up-to-date and to reduce language and literacy barriers, which could have influenced recruitment rates.

### Sampling

Data were collected across 35 sampling points (postcode sectors), which were randomly selected from a larger list maintained by Ethnic Focus of 370 postcode sectors in England with varying concentrations of ethnic minority groups according to census data. Sampling points were inspected for representation of high (10+ %), medium (7–9%), and low (5–7%) concentrations of ethnic minority residents, and properties within each sampling point were visited by a multi-lingual interviewer to determine whether eligible adults lived in the household based on age, gender, and ethnicity. Three call back visits to interview an eligible participant were made at a different time of day and week to the original call before considering them as a non-responder. No incentives were offered for study participation. The study was considered exempt from ethics approval under the UCL Research Ethics Committee Guidelines because participants were not considered to constitute a vulnerable group, participation was not deemed to cause undue stress or anxiety, and anonymous survey and interview procedures were used.

### Materials

Although data come from a wider study on cervical cancer, all items used for the present analyses were about cancer in general, not specifically cervical cancer. Women completed closed questions with a multi-lingual, female interviewer. Interviewers were competent in Gujarati, Hindi/Urdu, Syletti, French, Bangla, Punjabi, English, and Somali.

Interview materials were extensively piloted. First, the English language questionnaire was piloted with eight English-speaking women from Caribbean, African, and Asian backgrounds for comprehension of the questions. The questionnaire was then translated into the languages most commonly spoken by the target groups: Bengali, Gujarati, Hindi, Punjabi, Somali, and Urdu. After translation, questions were checked for consistent meaning by six bilingual researchers (Bengali, Urdu, Hindi, Punjabi, and Gujarati; we could not find a bilingual researcher for Somali), and interviewers were provided with an instruction sheet to clarify any items for which the meaning could be misinterpreted. Respondents could choose their preferred language from the seven languages in which the questionnaire was provided.

#### Cancer fear

Cancer fear was assessed using three items adapted from Berrenberg's Cancer Attitude Inventory (Berrenberg, 1991): ‘Of all diseases, I am most afraid of cancer' (greatest fear), ‘I worry a lot about cancer' (worry), and ‘It makes me uncomfortable to think about cancer' (discomfort). In a previous study (Vrinten *et al*, 2014), participants from ethnic minority backgrounds were more likely to endorse all three items than White British participants, but differences by ethnicity could not be explored further owing to the small number of ethnic minorities in that sample. Inter-item correlations in this study were moderate (ranging from 0.35 to 0.42), suggesting that the items tap different aspects of cancer fear (Vrinten *et al*, 2014). They were therefore analysed separately. All three items were measured on a 5-point Likert scale and dichotomised into ‘No' (0: ‘strongly disagree', ‘disagree', and ‘not sure') *vs* ‘Yes' (1: ‘agree' and ‘strongly agree').

#### Cancer fatalism

Cancer fatalism was measured using two items from the Powe Fatalism Inventory (Powe, 1995). One item assessed the belief that cancer is predetermined: ‘If someone is meant to get cancer, they will get it no matter what they do' (predetermination). The second item assessed the belief that cancer is incurable: ‘If someone has cancer, it is already too late to get treated' (incurability). The inter-item correlation for the entire sample was significant but small (*r*=0.19, *P*<0.001), indicating that these items tap into different components of cancer fatalism, and they were therefore analysed separately. Responses were made on a 5-point scale (0 ‘strongly disagree' to 4 ‘strongly agree') and were dichotomised into ‘No' *vs* ‘Yes', analogous to the cancer fear items.

#### Ethnicity, language, migration status, and literacy

Ethnicity was self-reported and assessed using a question from the 2011 census with 18 options (Office for National Statistics, 2011), but women were only included if they selected one of the six preset quotas. Migration status and ability to speak English were assessed using questions from the Office for National Statistics (2011) Census household questionnaire for England. Migration status was computed using date of birth, country of birth, and date of most recent arrival to live in the United Kingdom. We assessed health literacy using the question ‘How easy do you find it to understand leaflets and letters about your health?' (response options: ‘very easy', ‘fairly easy', fairly difficult', ‘very difficult') adapted from the European Health Literacy Project (HLS-EU Consortium, 2012).

#### General fatalism

General fatalism refers to the belief that events in life are determined by fate and this was assessed with a four-item measure (Jacobson, 1999), used in previous cancer-related studies (Lyratzopoulos *et al*, 2015). These questions were completed at the beginning of the survey before any cancer-specific questions were asked. Responses were made on a 5-point scale (0 ‘strongly disagree' to 4 ‘strongly agree') and a sum score was calculated (potential range: 0–16). Scale reliability in this sample was acceptable (Cronbach's alpha 0.61). Because of heterogeneity of variances between the ethnic groups and for ease of interpretation, fatalism scores were dichotomised according to the median of the overall sample (scores ⩾9 indicated high fatalism, scores <9 indicated low fatalism).

#### Sociodemographic factors

Data on age, educational qualifications, and marital status were assessed using 2011 census questions (Office for National Statistics, 2011). Age was categorised into three groups: ‘30–40', ‘41–50', and ‘51–60'. Educational level was categorised into ‘no formal qualifications', ‘some education', ‘degree level education', and ‘other'. Marital status was dichotomised into ‘married or cohabiting' *vs* ‘not married' (i.e., single/widowed/divorced).

### Analysis

Chi-square tests were used to explore differences in cancer fear and fatalism across all six ethnic groups using dichotomised variables and between the White British group and each of the ethnic groups separately. Focussing on Black, Asian, and Minority Ethnic (BAME) women only, we used logistic regression analyses to explore the role of ethnicity, migration status, ability to speak English, health literacy, and general fatalism on cancer fear and fatalism. Analyses were adjusted for sociodemographic differences (age, education, marital status, and ethnicity). We also conducted a sensitivity analysis by excluding all those who responded ‘not sure' to the cancer fear and cancer fatalism items and comparing those who disagreed and strongly disagreed with those who agreed and strongly agreed to these items. All analyses were carried out using SPSS 22.0 (IBM Corp., Armonk, NY, USA) and an alpha level of *P*<0.05 to indicate significance.

## Results

### Sample characteristics

In total, 1116 women were approached to obtain 720 completed surveys (response rate 64.5%). Of these, 52 women (7.2%) were excluded from the analyses because of a self-reported diagnosis of cancer (13 White British, 9 Caribbean, 6 African, 8 Indian, 8 Pakistani, 8 Bangladeshi). This left a sample of 668 women, of whom 561 (84.0%) were of non-White British ethnicity. Demographic characteristics of the sample are presented in Table 1. There were differences between ethnic groups in the level of education and marital status, reflecting differences in the population as a whole.

All of the White British women and about half of the Caribbean women were born in the United Kingdom with a further 43% of Caribbean women migrating to the United Kingdom as a child. For the other ethnic groups, about half to two-thirds had migrated to the United Kingdom as an adult. All of the White British and Caribbean women and most of the African women (64%) had English as their main language, compared with one-third of Indian and Pakistani women (35% and 34%, respectively), and a quarter of Bangladeshi women (26%). Again, these percentages reflect the census data for this age range. All Caribbean (100%) and nearly all White British women (93%) found it easy to understand letters and leaflets about health, *vs* two-thirds of African (68%), and less than half of Indian, Pakistani, and Bangladeshi women (45, 44, and 28%, respectively). There were large ethnic differences in general fatalism: few White British (11%), Caribbean (17%), or African women (27%) scored high on general fatalism, but most Indian (76%), Pakistani (81%), and Bangladeshi women (82%) did.

### Ethnic differences in cancer fear and cancer fatalism

Inter-item correlations for cancer fear and cancer fatalism are shown in Table 2. There was a strong, positive correlation between having cancer as the greatest health fear and cancer worry (*r*=0.61). Other items were not at all, or only weakly, correlated (Pearson's *r* 0.01–0.23).

Univariate analyses showed that there were ethnic differences in cancer fear as indexed by having cancer as the greatest health fear (*P*<0.05) and worrying about cancer (*P*<0.001), with more Indian women having cancer as the greatest health fear than White British women and African women being more worried about cancer than White women (see Table 3). There were no ethnic differences in discomfort when thinking about cancer (*P*=0.14). Bangladeshi women were least likely to fear cancer more than other diseases (15%), followed by White British and Caribbean (19% and 22%, respectively), African and Pakistani (25% and 27%, respectively), and Indian women (34%). A similar pattern was observed for cancer worry: percentages of worry were lowest in the Bangladeshi, Caribbean, and White British groups (9, 14, and 16%, respectively), and highest in Indian and African women (21% and 33%, respectively).

BAME women were more fatalistic about cancer than White British women. Very few White British women (6%) believed that a diagnosis of cancer was predetermined, *vs* 11% of African women, 23% of Caribbean women, and at least half of Indian, Pakistani, and Bangladeshi women (50, 52, and 63%, respectively; *P*<0.001). Furthermore, White British women did not believe that cancer is incurable, but a quarter to a third of all BAME women held this belief (*P*<0.001; see Table 3).

### Predictors of cancer fear

We examined associations between migration status, ability to speak English, health literacy and general fatalism, and cancer fear and cancer fatalism in BAME women (*n*=561), using the Caribbean group as the reference category because they were most similar to White British women. The results of the unadjusted and adjusted analyses were very similar, so only the adjusted analyses are presented in Table 4. After adjusting for age, education, and marital status, Indian women were more likely to fear cancer more than other diseases compared with Caribbean women (34% *vs* 22%), African women were more worried about cancer (33% *vs* 14%), and Bangladeshi women were less likely to feel uncomfortable at the thought of cancer (18% *vs* 23%), with no significant differences for the other groups compared with the Caribbean group.

When also adjusting for these ethnic differences, those who had migrated to the United Kingdom as an adult were less likely to have cancer as their greatest health fear (15%, *vs* 37% of those born in the United Kingdom), with similar findings for those whose main language was not English (14–21% *vs* 32%) and who found letters and leaflets about health difficult to understand (6–22% *vs* 33%). The same pattern was found for worrying about cancer a lot (migration: 15% *vs* 24% English as a main language: 10–20% *vs* 25% understanding health leaflets: 6–22% *vs* 24%). However, we found the opposite effect for feeling uncomfortable when thinking about cancer: those who were not born in the United Kingdom (30% *vs* 15%), who did not speak English as their main language (31–38% *vs* 19%), and who had some difficulty understanding health leaflets (35–41% *vs* 18%) were more likely to be uncomfortable about cancer. General fatalism marginally decreased feeling particularly fearful of cancer *vs* other diseases (21% *vs* 29%) but was not associated with the other two cancer fear measures.

### Predictors of cancer fatalism

Adjusting for differences in age, education, and marital status, African women were less likely to believe that a diagnosis of cancer is predetermined compared with Caribbean women (11% *vs* 23% see Table 4), while Pakistani and Bangladeshi women were more likely to hold this belief (52% and 63%, respectively). Bangladeshi women were also more likely to believe that cancer is incurable (38% *vs* 26%). When also adjusting for these ethnic differences, those who were not born in the United Kingdom (37–56% *vs* 16%), whose main language was not English (59–68% *vs* 16%), and who had difficulty understanding health leaflets (43–74% *vs* 16%) were more likely to feel that a diagnosis of cancer is predetermined. The belief that cancer is incurable was not associated with any of these variables. Similarly, general fatalism was positively associated with believing that cancer is predetermined (56% *vs* 19%) but not with the belief that cancer is incurable.

### Sensitivity analysis

Excluding those who responded ‘not sure' did not make much difference to the endorsement rates of the cancer fear items by each ethnic minority group (results not shown) or for the belief that a diagnosis of cancer is predetermined. However, endorsement rates for the belief that it is too late to get treated if cancer is found increased across all ethnic minority groups, likely owing to the high numbers of women who responded ‘not sure' to this item (15% of Caribbean women, 28% of African, 36% of Indian, 41% of Pakistani, and 34% of Bangladeshi women were excluded). After excluding ‘not sures', endorsement rates for cancer being incurable were 31% for Caribbean, 49% for African, 53% for Indian, 46% for Pakistani, and 58% for Bangladeshi women. Rerunning the logistic regression analysis on this smaller sample did not change the direction of the associations between ethnicity and cancer fear or the belief that a diagnosis of cancer is predetermined. However, women from all ethnic backgrounds were more likely than Caribbean women to believe that cancer is incurable (African OR=2.66, 95% CI 1.38–5.15; Indian OR=2.82, 95% CI 1.35–5.87; Pakistani OR=2.18, 95% CI 1.03–4.58, Bangladeshi OR=3.85, 95% CI 1.79–8.29; full results available from the first author upon request).

## Discussion

This study suggests that cancer remains widely feared, and that cancer fear and fatalism vary across BAME groups in the United Kingdom. Caribbean and Pakistani women show similar levels of cancer fear to White British women, while Bangladeshi women are less afraid of cancer, and African and Indian women are more afraid. Among BAME women, those who had migrated to the United Kingdom as adults and did not speak English very well were less likely to worry about cancer or fear it more than other diseases, but more likely to feel uncomfortable about cancer. Cancer fatalism was higher in all BAME women than White British women. Beliefs about cancer predetermination were associated with lower acculturation and a more fatalistic outlook on life in general, but these factors were unrelated to the belief that cancer is incurable.

The similar levels of cancer fear for Caribbean and White British women could reflect the fact that Caribbean women are more acculturated: most had lived in the United Kingdom since childhood and used English as their main language. Bangladeshi women also formed an exception: they scored consistently lower than White British women on all measures of cancer fear. Some authors suggest that both high and low levels of fear can impede cancer screening uptake (Andersen *et al*, 2003; Champion *et al*, 2004; Consedine *et al*, 2006), owing to a lack of threat (at low levels of fear) or paralysing fear (high levels). Asian women (who in our sample scored both the highest and the lowest on cancer fear) have been shown to have high rates of non-attendance at cervical cancer screening (Marlow *et al*, 2015). Future research should explore whether this is due to particularly high or low levels of cancer fear in these groups.

We found that those who were less acculturated (i.e., had migrated to the United Kingdom as adults, did not speak English, or found health letters and leaflets difficult to understand) were less likely to worry about cancer or be particularly fearful of the disease. One possible explanation for this finding comes from qualitative studies that suggest that cancer fears in less acculturated samples may be more reflective of the country of origin and that other threats may be more prominent in particular countries (Buki *et al*, 2004; Marlow *et al*, 2014). Cancer is also sometimes seen as a ‘Western disease' (Jackson *et al*, 2000; Buki *et al*, 2004; Kwok, 2005), which could contribute to lower levels of cancer worry, especially when compared with other illnesses. Alternatively, greater exposure to cancer awareness campaigns in the host country (i.e., the United Kingdom) may also increase cancer worries and feelings of susceptibility to cancer, particularly in those who are better equipped to engage with these campaigns (i.e., the more acculturated), raising important questions about the origins of cancer fear.

Our findings have implications for public health, in particular cancer awareness and early diagnosis campaigns. Remarkably, no White British women believed that cancer was incurable, but a significant proportion of BAME women endorsed this belief, regardless of acculturation and general fatalism, raising questions about the origins of this belief. One possibility is that previous encounters with cancer influence beliefs about survivability and that these are particularly bad in ethnic minorities. In some cultures, cancer is considered a ‘taboo', and services may therefore not be accessed until a late stage in the disease when it is no longer curable (Randhawa and Owens, 2004; Opoku, 2012; Granado *et al*, 2014). This idea is supported by findings that some BAME groups have worse cancer survival for certain cancers, which is partially attributable to an advanced stage at diagnosis (Jack *et al*, 2009; National Cancer Intelligence Network, 2009). Regardless of the origins of this belief, our findings suggest that messages about increased cancer survival are not reaching all BAME women, which was also reflected by the large proportion of women who were excluded from the sensitivity analyses because they were ‘not sure' about whether cancer is curable (15–34%). It is likely that the belief that cancer is incurable influences BAME women's engagement with cancer awareness campaigns and early detection services. If so, it is important that these disparities are addressed.

Examining the effects of these ethnic differences in fear and fatalism on early detection of cancer and screening uptake in specific ethnic groups could help inform more targeted campaigns. For example, Bangladeshi women not only had the lowest levels of cancer fear and worry but also the highest endorsement levels for fatalistic beliefs about cancer. Low levels of cancer worry could be due to a perceived lack of susceptibility to cancer. In that case, campaigns targeted at Bangladeshi women should address the combination of low perceived susceptibility and beliefs about the incurability and predetermination of cancer to encourage health-protective behaviour in this group. African and Indian women, and, to a lesser extent, Pakistani women, on the other hand, experienced high levels of cancer fear together with high endorsement of fatalistic beliefs about cancer. Campaigns to promote cancer early detection and screening in these groups may need to focus on addressing fatalistic beliefs about cancer in combination with reducing the high, possibly debilitating, levels of fear associated with cancer in these groups. There are also stark differences by acculturation, regardless of ethnicity: those who migrated more recently, whose main language is not English, and who have difficulty understanding health information are more likely to believe that cancer is predetermined and to feel uncomfortable discussing cancer. This may constitute an important barrier towards engagement with cancer awareness and early detection campaigns that may need to be addressed in these groups.

This study also has implications for future research. The two fatalistic beliefs about cancer used in this study (predetermination and incurability) are not usually distinguished; even if a measure includes both, such as the Powe Fatalism Index (Powe, 1995), results are usually not reported by subscale. However, it may be important to examine these beliefs separately, especially when exploring their contributions to non-uptake of cancer screening, as this may have practical consequences for the design of public health campaigns. For example, the belief that cancer is incurable may be much easier to address than the belief that a cancer diagnosis is predetermined, especially if the latter belief stems from a belief that events in life are generally predetermined.

We found a small but significant, negative correlation between cancer worry and cancer predetermination, which could suggest that the belief in cancer predetermination may partially protect against cancer worry. Negative emotional states such as cancer worry tend to be regulated (Consedine *et al*, 2004a), and a belief that a diagnosis of cancer is predetermined may make worrying redundant. Empirical support for this notion comes from a Japanese study that found that lung cancer patients who were ‘fatalistic' (defined as being accepting that they had no control over their prognosis) were less likely to suffer from significant anxiety (Shimizu *et al*, 2015), again suggesting that there is a possible emotional benefit of predetermination beliefs. However, most studies on cancer fear and fatalism, including the current one, are cross-sectional. Thus conclusions about the causal relationship of fear and fatalism cannot be drawn. Longitudinal studies would be needed to create a better understanding of the causal pathways between cancer fear and fatalism.

Another implication for future research comes from our finding that correlations between the cancer fear and fatalism items were generally modest and that their associations with the acculturation and fatalism variables varied. This supports the idea that the items tap into different components of fear and fatalism. Previous research has shown that the sociodemographic associations and the effects of the three cancer fear components on colorectal cancer screening uptake vary (Vrinten *et al*, 2014, 2015), and future studies should address whether this is similar for the two fatalistic beliefs.

Our study has several limitations. It was part of a larger study that was not designed primarily to look at cancer fear and cancer fatalism in ethnic minority women. The sample was limited to women, and quotas for ethnicity were used to ensure equal representation of ethnicities across the sample; thus the sample was not representative of the UK population or the UK ethnic minority population. Care was taken to preserve the meaning when translating the surveys into the languages most commonly spoken by the target groups, but there may have been slight cultural differences in meaning between the translations. The components of cancer fear and cancer fatalism were measured with single items, which may have limited the reliability of these measures. The measure for educational attainment was based on the UK 2011 Census measure, which asks about UK qualifications. Respondents with foreign qualifications are encouraged to map their foreign qualifications onto their equivalents within the UK educational system, but the high proportions of Indian, Pakistani, and Bangladeshi women reporting ‘other' types of qualifications may indicate that these groups found it difficult to do so. This is part of a wider problem of accurately measuring educational attainment or socioeconomic status in ethnic minority groups, especially women. Future studies may want to consider including measures that do not require mapping onto the UK educational system, such as age at which the respondent left school. Finally, marital status was adjusted for because previous research has shown that those who are not married tend to be more fatalistic and fearful about cancer (Hall *et al*, 2008; Vrinten *et al*, 2014), but we did not explore whether these associations were dependent on the ethnicity of the partner.

## Conclusion

Cancer fear and fatalism are generally more prevalent among BAME women than White British women and are influenced by migration status, language spoken, and fatalistic beliefs about life in general. Those who are less acculturated are less likely to worry about cancer but are more likely to feel uncomfortable about cancer and to believe that a cancer diagnosis is a matter of fate. These beliefs may affect their engagement with cancer early detection campaigns and participation in cancer screening.

## Figures and Tables

**Table 1:** Characteristics of the sample (*N*=668)

	**Overall**	**White British**	**Caribbean**	**African**	**Indian**	**Pakistani**	**Bangladeshi**	**Significance**
*N* (%)	668 (100)	107 (16.0)	111 (16.6)	114 (17.1)	112 (16.8)	112 (16.8)	112 (16.8)	
**Age, years**								
30–40	284 (42.5)	39 (36.4)	45 (40.5)	52 (45.6)	41 (36.6)	51 (45.5)	56 (50.0)	*χ*^2^(10)=10.50, *P*=0.40
41–50	241 (36.1)	39 (36.4)	40 (36.0)	44 (38.6)	44 (39.3)	36 (32.1)	38 (33.9)	
51–60	143 (21.4)	29 (27.1)	26 (23.4)	18 (15.8)	27 (24.1)	25 (22.3)	18 (16.1)	
**Education**								
No formal qualification	94 (14.1)	0 (0)	25 (22.5)	21 (18.4)	0 (0)	28 (25.0)	20 (17.9)	*χ*^2^(15)=322.62, *P*<0.001
Some	305 (45.7)	94 (87.9)	54 (48.6)	72 (63.2)	20 (17.9)	33 (29.5)	32 (28.6)	
Degree	124 (18.6)	13 (12.1)	32 (28.8)	21 (18.4)	35 (31.3)	19 (17.0)	4 (3.6)	
Other	145 (21.7)	0 (0)	0 (0)	0 (0)	57 (50.9)	32 (28.6)	56 (50.0)	
**Marital status**								
Married or cohabiting	477 (71.4)	66 (61.7)	40 (36.0)	70 (61.4)	91 (81.3)	103 (92.0)	107 (95.5)	*χ*^2^(5)=138.99, *P*<0.001
Not married	191 (28.6)	41 (38.3)	71 (64.0)	44 (38.6)	21 (18.8)	9 (8.0)	5 (4.5)	
**Migration status**								
Born in the United Kingdom	287 (43.0)	107 (100)	53 (47.7)	24 (21.1)	38 (33.9)	36 (32.1)	29 (25.9)	*χ*^2^(10)=311.07, *P*<0.001
<18 years	102 (15.3)	0 (0)	48 (43.2)	18 (15.8)	8 (7.1)	23 (20.5)	5 (4.5)	
>18 years	279 (41.8)	0 (0)	10 (9.0)	72 (63.2)	66 (58.9)	53 (47.3)	78 (69.6)	
**Ability to speak English**								
Main language	394 (59.0)	107 (100)	111 (100)	73 (64.0)	38 (33.9)	36 (32.1)	29 (25.9)	*χ*^2^(10)=286.75, *P*<0.001
Well/very well	85 (12.7)	0 (0)	0 (0)	24 (21.1)	22 (19.6)	22 (19.6)	17 (15.2)	
Not well/not at all	189 (28.3)	0 (0)	0 (0)	17 (14.9)	52 (46.4)	54 (48.2)	66 (58.9)	
**Understanding leaflets or letters about health**								
Very easy	322 (48.2)	58 (54.2)	108 (97.3)	53 (46.5)	38 (33.9)	36 (32.1)	29 (25.9)	*χ*^2^(15)=311.76, *P*<0.001
Fairly easy	95 (14.2)	41 (38.3)	3 (2.7)	24 (21.1)	12 (10.7)	13 (11.6)	2 (1.8)	
Fairly difficult	196 (29.3)	6 (5.6)	0 (0.0)	35 (30.7)	46 (41.1)	58 (51.8)	51 (45.5)	
Very difficult	55 (8.3)	2 (1.9)	0 (0.0)	2 (1.8)	16 (14.3)	5 (4.5)	30 (26.8)	
**General fatalism**								
Low	338 (50.6)	95 (88.8)	92 (82.9)	83 (72.8)	27 (24.1)	21 (18.8)	20 (17.9)	*χ*^2^(5)=256.12, *P*<0.001
High	330 (49.4)	12 (11.2)	19 (17.1)	31 (27.2)	85 (75.9)	91 (81.2)	92 (82.1)	

**Table 2:** Pearson's correlations between cancer fear and fatalism across all ethnicities, before item dichotomisation (*N*=668)

	**Cancer fear Greatest fear**	**Cancer worry**	**Cancer discomfort**	**Cancer fatalism Predetermination**
**Cancer fear**				
Cancer worry	0.61***			
Cancer discomfort	0.01	0.12**		
**Cancer fatalism**				
Predetermination	−0.05	−0.16***	0.15***	
Incurability	0.08*	0.05	0.03	0.23***

Note: *P<0.05, **P<0.01, ***P<0.001.

**Table 3:** Ethnic differences in cancer fear and fatalism (*N*=668)^a^

**N (% agree)**	**Overall**	**White British**	**Caribbean**	**African**	**Indian**	**Pakistani**	**Bangladeshi**	**Significance**
**Cancer fear**								
**Cancer as greatest health fear**								
No	510 (76.3)	87 (81.3)	87 (78.4)	85 (74.6)	**74 (66.1)**	82 (73.2)	95 (84.8)	*χ*^*2*^*=13.5, P*<0.*05*
Yes	158 (23.7)	20 (18.7)	24 (21.6)	29 (25.4)	**38 (33.9)**	30 (26.8)	17 (15.2)	
**Cancer worry**								
No	544 (81.4)	90 (84.1)	95 (85.6)	**76 (66.7)**	89 (79.5)	92 (82.1)	102 (91.1)	*χ*^*2*^*=25.4, P*<0.*001*
Yes	124 (18.6)	17 (15.9)	16 (14.4)	**38 (33.3)**	23 (20.5)	20 (17.9)	10 (8.9)	
**Cancer discomfort**								
No	498 (74.6)	81 (75.7)	86 (77.5)	79 (69.3)	76 (67.9)	84 (75.0)	92 (82.1)	*χ*^*2*^*=8.3, P*=0.*14*
Yes	170 (25.4)	26 (24.3)	25 (22.5)	35 (30.7)	36 (32.1)	28 (25.0)	20 (17.9)	
**Cancer fatalism**								
**Predetermination**								
No	439 (65.7)	101 (94.4)	**85 (76.6)**	101 (88.6)	**56 (50.0)**	**54 (48.2)**	**42 (37.5)**	*χ*^*2*^*=138.4, P*<0.*001*
Yes	229 (34.3)	6 (5.6)	**26 (23.4)**	13 (11.4)	**56 (50.0)**	**58 (51.8)**	**70 (62.5)**	
**Incurability**								
No	488 (73.1)	107 (100)	**82 (73.9)**	**74 (64.9)**	**74 (66.1)**	**82 (73.2)**	**69 (61.6)**	*χ*^*2*^*=53.6, P*<0.*001*
Yes	180 (26.9)	0 (0)	**29 (26.1)**	**40 (35.1)**	**38 (33.9)**	**30 (26.8)**	**43 (38.4)**	

a Ethnic groups that are significantly different from the White British group are in bold.

**Table 4:** Adjusted logistic regression analyses of cancer fear and cancer fatalism in BAME women (*N*=561)

	**Cancer fear**						**Cancer fatalism**			
	**Greatest fear**		**Worry**		**Discomfort**		**Predetermination**		**Incurability**	
	**%**	**OR (95% CI)**	**%**	**OR (95% CI)**	**%**	**OR (95% CI)**	**%**	**OR (95% CI)**	**%**	**OR (95% CI)**
**Ethnicity**^a^										
Caribbean	21.6	1.00	14.4	1.00	22.5	1.00	23.4	1.00	26.1	1.00
African	25.4	1.17 (0.61–2.26)	33.3	**2.99 (1.50–5.96)**	30.7	1.48 (0.79–2.75)	11.4	**0.38 (0.18–0.80)**	35.1	1.74 (0.96–3.16)
Indian	33.9	**2.40 (1.20–4.81)**	20.5	1.91 (0.87–4.22)	32.1	1.00 (0.48–2.06)	50.0	1.87 (0.93–3.76)	33.9	1.70 (0.88–3.29)
Pakistani	26.8	1.54 (0.77–3.09)	17.9	1.54 (0.70–3.42)	25.0	0.83 (0.41–1.70)	51.8	**2.14 (1.09–4.19)**	26.8	1.14 (0.59–2.23)
Bangladeshi	15.2	1.00 (0.46–2.18)	8.9	0.88 (0.35–2.22)	17.9	**0.45 (0.20–0.99)**	62.5	**2.71 (1.34–5.49)**	38.4	**2.21 (1.12–4.36)**
**Migration status**^b^										
Born in the UK	37.2	1.00	24.4	1.00	15.0	1.00	16.1	1.00	31.1	1.00
Under 18	27.5	0.59 (0.31–1.11)	20.6	0.63 (0.32–1.25)	30.4	**1.97 (1.02–3.81)**	37.3	**4.30 (2.10–8.80)**	36.3	1.18 (0.65–2.15)
Over 18	15.4	**0.27 (0.13–0.54)**	15.1	**0.40 (0.19–0.82)**	30.8	1.60 (0.81–3.19)	55.9	**7.29 (3.48–15.26)**	31.2	0.66 (0.35–1.23)
**Speak English**^b^										
Main language	32.4	1.00	24.7	1.00	18.5	1.00	15.7	1.00	30.3	1.00
Well/very well	21.2	**0.31 (0.15–0.63)**	20.0	**0.45 (0.22–0.92)**	37.6	**3.03 (1.55–5.91)**	58.8	**14.42 (6.50–31.98)**	35.3	0.99 (0.53–1.84)
Not well/not at all	14.3	**0.26 (0.12–0.59)**	10.1	**0.29 (0.12–0.68)**	31.2	**2.46 (1.17–5.18)**	67.7	**14.95 (6.42–34.83)**	33.3	0.97 (0.50–1.89)
**Understand health letters and leaflets**^b^										
Very easy	33.3	1.00	24.2	1.00	17.8	1.00	16.3	1.00	30.3	1.00
Fairly easy	22.2	**0.26 (0.12–0.59)**	22.2	**0.38 (0.17–0.83)**	40.7	**2.83 (1.36–5.87)**	42.6	**9.87 (4.09–23.80)**	42.6	1.29 (0.65–2.57)
Fairly difficult	18.4	**0.30 (0.15–0.62)**	14.7	**0.37 (0.18–0.78)**	34.7	**2.18 (1.07–4.45)**	62.1	**14.86 (6.40–34.50)**	29.5	0.67 (0.35–1.28)
Very difficult	5.7	**0.11 (0.03–0.43)**	5.7	**0.21 (0.05–0.88)**	17.0	0.88 (0.31–2.51)	73.6	**17.27 (6.00–49.68)**	39.6	0.98 (0.41–2.36)
**Fatalism**^b^										
Low	28.8	1.00	22.6	1.00	21.8	1.00	18.9	1.00	34.2	1.00
High	21.4	**0.60 (0.36–1.00)**	16.4	0.84 (0.49–1.44)	28.6	1.42 (0.84–2.38)	55.7	**2.29 (1.40–3.77)**	30.5	0.63 (0.39–1.01)

Abbreviations: BAME=Black, Asian, and Minority Ethnic; CI=confidence interval; OR=odds ratio. Significant values are in bold.

a Adjusted for age, education, and marital status.

b Adjusted for age, education, marital status, and ethnicity.
